# Supplementary material for: Relationship between Preoperative Pyuria and Bacille Calmette-Guerin Treatment in Intravesical Recurrence after Transurethral Resection of High-Risk, Non-Muscle Invasive, Bladder Carcinoma: A Retrospective Study of Human Data
Source: Cancers (Basel). 2023 Mar 7;15(6):1638. doi: 10.3390/cancers15061638 (PMC10046501; doi:10.3390/cancers15061638)
Supplement: Supplementary file 1 [file cancers-15-01638-s001.zip › cancers-2270513-supplementary.pdf]

# Supplementary Materials: Relationship between preoperative pyuria and bacille Calmette-Guerin treatment in intravesical recurrence after transurethral resection of high-risk, non-muscle invasive, bladder carcinoma: A retrospective study of human data

**Ryo Tsukamoto, Tomokazu Sazuka, Yoshinori Hattori, Hiroaki Sato, Takayuki Arai, Yusuke Goto, Yusuke Imamura, Shinichi Sakamoto and Tomohiko Ichikawa**

**Table S1.** BCG treated patients subdivided according to the presence of preoperative pyuria.

| | | **Total n=179** | **Pyuria present (n=45)** | **Pyuria absent (n=134)** | **P-value** |
|---|---|---|---|---|---|
| Age | Mean (range) | 73(38-89) | 75(56-89) | 73(38-87) | 0.0841 |
| Sex | Male | 141(78.8) | 35(77.8) | 106(79.1) | 0.8506 |
| | Female | 38(21.2) | 10(22.2) | 28(20.9) | |
| Preoperative cytology | Pogitive | 55(30.7) | 16(35.6) | 39(29.1) | 0.417 |
| | Negative | 124(69.3) | 29(64.4) | 95(70.9) | |
| Grade | High | 160(89.4) | 39(86.7) | 121(90.3) | 0.4938 |
| | Low | 19(10.6) | 6(13.3) | 13(9.7) | |
| Pathological T stage, n (%) | T1 | 64(35.8) | 20(44.4) | 44(32.8) | 0.1598 |
| | Others | 115(64.2) | 25(55.6) | 90(67.2) | |
| Concomitant CIS | Present | 32(11.6) | 8(17.8) | 25(18.7) | 0.8938 |
| | Absent | 147(88.4) | 37(82.2) | 109(81.3) | |

**Table S2.** non-BCG treated patients subdivided according to the presence of preoperative pyuria.

| | | **Total n=245** | **Pyuria present (n=38)** | **Pyuria absent (n=207)** | **P-value** |
|---|---|---|---|---|---|
| Age | Mean (range) | 72(39-94) | 71(50-90) | 75(39-94) | 0.3462 |
| Sex | Male | 195(79.6) | 23(60.5) | 172(83.1) | 0.0015 |
| | Female | 50(20.4) | 15(39.5) | 35(16.9) | |
| Preoperative cytology | Pogitive | 217(88.6) | 3(7.9) | 25(12.1) | 0.4563 |
| | Negative | 28(21.4) | 35(92.1) | 182(87.9) | |
| Grade | High | 165(67.3) | 30(78.9) | 135(65.2) | 0.0971 |
| | Low | 80(32.7) | 8(21.1) | 72(34.8) | |
| Pathological T stage, n (%) | T1 | 45(18.4) | 12(31.6) | 33(15.9) | 0.0221 |
| | Others | 200(81.6) | 26(68.4) | 174(84.1) | |
| Concomitant CIS | Present | 15(61.2) | 1(2.6) | 15(7.2) | 0.2899 |
| | Absent | 230(38.8) | 37(97.4) | 192(92.8) | |

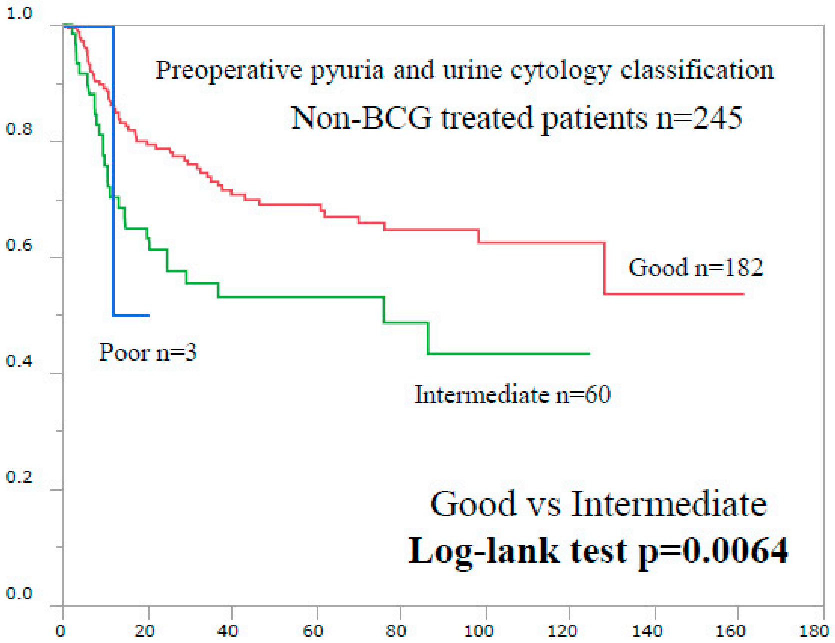


| | | | | | | | |
|---|---|---|---|---|---|---|---|
| Good | 182 | 125 | 91 | 67 | 48 | 28 | 10 |
| Intermediate | 60 | 36 | 24 | 17 | 12 | 3 | 2 |
| Poor | 3 | 1 | | | | | |

**Figure S1.** Intravesical recurrence-free survival rates after trans-urethral resection of bladder tumors in non-BCG treated patients according to preoperative pyuria and cytology status. Low risk: cytology (–) and pyuria (–); intermediate risk: cytology (+) and pyuria (–)/cytology (–) and pyuria (+); high risk: cytology (+) and pyuria (+). Rates were estimated using the Kaplan–Meier method.
